# Enrichment of Human-Computer Interaction in Brain-Computer Interfaces via Virtual Environments

**DOI:** 10.1155/2017/6076913

**Published:** 2017-11-29

**Authors:** Alonso-Valerdi Luz María, Mercado-García Víctor Rodrigo

**Affiliations:** Escuela de Ingeniería y Ciencias, Tecnológico de Monterrey, Eugenio Garza Sada 2501, 64849 Monterrey, NL, Mexico

## Abstract

Tridimensional representations stimulate cognitive processes that are the core and foundation of human-computer interaction (HCI). Those cognitive processes take place while a user navigates and explores a virtual environment (VE) and are mainly related to spatial memory storage, attention, and perception. VEs have many distinctive features (e.g., involvement, immersion, and presence) that can significantly improve HCI in highly demanding and interactive systems such as brain-computer interfaces (BCI). BCI is as a nonmuscular communication channel that attempts to reestablish the interaction between an individual and his/her environment. Although BCI research started in the sixties, this technology is not efficient or reliable yet for everyone at any time. Over the past few years, researchers have argued that main BCI flaws could be associated with HCI issues. The evidence presented thus far shows that VEs can (1) set out working environmental conditions, (2) maximize the efficiency of BCI control panels, (3) implement navigation systems based not only on user intentions but also on user emotions, and (4) regulate user mental state to increase the differentiation between control and noncontrol modalities.

## 1. Introduction

**Figure 1:**
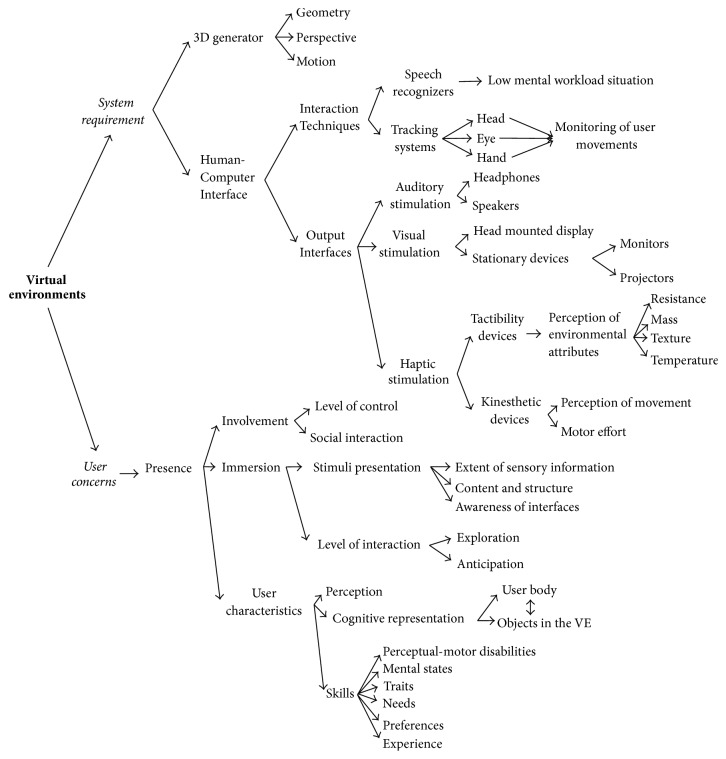
Structure of a virtual environment on the basis of two key elements: system requirements and user concerns.

Brain-Computer Interfaces (BCI) are systems that attempt to establish communication between the human brain and a computer in order to replace the natural connection between central nervous system (CNS) and musculoskeletal system. The interest on BCI research has been greatly increased due to a wide variety of applications, including neurorehabilitation, robotic devices, exoeskeletons, and domotic systems. Although BCI research started in the sixties, this technology is not efficient or reliable yet for everyone at any time. Over the past few years, some researchers such as Fabien Lotte and Camille Jeunet have argued that main BCI flaws could be associated with human-computer interaction (HCI) issues [1–4]. As can be seen in Figure 1, virtual environments (VEs) have many distinctive features that can significantly improve HCI in highly demanding and interactive systems such as BCI. The present paper moves on to describe in greater detail five key points:

- Main characteristics of VEs (Section 2)
- How those characteristics can improve HCI (Section 3)
- How the improvement of HCI via VE may help to overcome several drawbacks of BCI systems (Section 4)
- Extensive revision of recent advances in the field (Section 4)
- Strong tendencies of this research area (Section 5).

## 2. Virtual Environments: System Requirements and User Concerns

People have an overall clear perception of their environment in spite of their limited sensory system. Owing to the extraordinary signal processing of the nervous system, which constantly updates human reactions, people can carry out complex activities. For example, a person is capable of recognizing and classifying a large number of sounds merged in a surrounding space. It is, therefore, a difficult task to develop VEs that generate synthetic visual, auditory, and haptic sensations, which could deceive human perception. A VE has two basic elements: system requirements and user concerns [5]. Figure 1 provides a summary of all the components encompassed under these two categories.

With respect to system requirements, a VE generally requires a 3D generator and a HCI. The 3D generator consists in modeling and animating 3D objects under the following criteria: (1)* geometry*, definition of the visual appearance, sound, odor, taste, and/or texture of each object in the VE; (2)* perspective*, spatial relationship between the geometry and the user; and (3)* motion*, geometrical changes in response to user actions and time progress. Regarding the HCI, there are* output interfaces* for stimulating the user senses and* interaction techniques* for decoding the user desires. The* output interface*s are classified as auditory, visual, and haptic devices. Auditory devices foster user awareness, and even the high quality sound can help in creating a more realistic and immersive experience. Headphones and speakers are the most commonly used auditory devices [6–9]. Visual devices allow users to see around, over, and under objects and also give users a stereoscopic vision of the VE [10–12]. They can be head-mounted devices or stationary devices such as monitors and projectors. Haptic systems are divided into tactibility and kinesthetic devices. Tactibility devices provide tactile feedback to perceive the attributes of the environment such as resistance, mass, texture, or temperature. Kinesthetic devices provide perception of movement or motor effort [13–15]. The* interaction techniques* refer to the mode of interacting with the VE. The common ones are graphical user interfaces, speech recognizers, and head/eye/hand tracking systems. Speech recognizers are suitable for low mental workload situations because humans tend to block their auditory channels under extreme workload situations. Tracking systems are position sensors that monitor the user movements in the VE. This allows the VE generator to render and display the VE from the user perspective, achieving the effect of physical immersion [13–15]. Some examples of tracking systems are as follows: (1) electromagnetic sensors to determine position and orientation, (2) mechanical sensors to simulate force effects, (3) optical sensors to determine 3D position, (4) ultrasonic sensors to calculate distances, and (5) inertial sensors to detect motion such as gyroscopic force, acceleration, or inclination.

In addition to the technological side, the human side of these cybersystems (or user concerns) must be also considered. User concerns are associated with the generation of a virtual world cognitively equivalent to the real one. The closest similarity between these two worlds takes place when users have the sense of being there. Users interact in and with the virtual space as if they were there; that is, they experience presence. Presence occurs when users feel immersed in the VE, feel capable of interacting with it, and have an interest in undertaking tasks. The three main aspects of presence are immersion, user characteristics, and involvement [16, 17]. Immersion is brought about when users perceive themselves to be enveloped by and included in the VE. The stimuli presentation and the level of interaction are the tools that a virtual system uses to have a good quality immersion. The stimuli presentation depends on three factors: (1) quality of immersion related to the extent of sensory information presented to VE users, (2) dramatic content and structure that are implemented in the VE, and (3) awareness of interfaces that distracts from the VE experience. The level of interaction is controlled by the possibility of exploring extensively the VE and the ability to predict and anticipate what will happen next [18, 19]. The virtual interaction is highly modified by individuals' characteristics, and because they cannot be controlled, they must be considered. User perception dynamically changes as users move through and interact with the VE, so this is the first psychological process to take into account. The cognitive representation of the VE is another important individual contribution, which captures the relation between the user body and the objects in the environment. Finally, user skills vary significantly across individuals, distorting the virtual interaction. Some instances of such skills are perceptual-motor abilities, mental states, traits, needs, preferences, and experience. Last but not least, the last element of VEs in terms of user concerns is involvement. The relation between the VE as a space and the individual body is called involvement. When the level of control that users have over the virtual sensor mechanisms is high, and their social interaction with the VE is good, users focus on the system suppressing possible constraints of the VE. As a result, users forget the real environment achieving a complete involvement [20].

## 3. Improvement of Human-Computer Interaction via Virtual Environments

As VEs rely on representing real-life traits, objects, and scenarios, 3D representations of objects and places augment user experience (UX), in comparison with 2D representations. Tridimensional representations stimulate cognitive processes that are the core and foundation of HCI. Those cognitive processes take place while the user navigates and explores the VE and are mainly related to spatial memory storage, attention, and perception. Even more important, such cognitive processes could be somehow modulated since VEs are designed according to both research goals and user needs [12]. In addition, VEs easily reach user engagement and UX, two desire factors in a proficient HCI. So far, VEs have been validated as an effective, safe, and motivating approach used to enhance the interaction between a user and a system [21].

VEs cannot, however, contribute to HCI by itself. User interaction in VEs could become sloppy, redundant, and frustrating. Along with a realistic and sophisticated design, VEs must be conceptualized and designed according to human factors and user characteristics.

## 4. Integration of Virtual Environments and Brain-Computer Interfaces

VEs have been widely used in BCI development to increase motivation and immersion, and a wide variety of scenarios have been proposed, from daily life situations to video games [12]. Several applications of VEs in BCI have included the control of virtual cars [22], navigations through virtual bars [21] or virtual flats [23], and walks through virtual streets [24]. One of the most common applications is in domotic systems. For example, a typical situation is to make an avatar to select and manipulate 3D virtual objects such as turning on/off lights, TVs, or lamps [25]. Other applications are wheelchair control, flying simulators [26], and virtual cities [27]. In sections that follow, BCI research is summarized, scientific relevance of BCI is discussed, current shortcomings of BCI are argued, the VE role in BCI research is justified, and a review of advances in the field is provided.

### 4.1. Brain-Computer Interfaces

**Figure 2:**
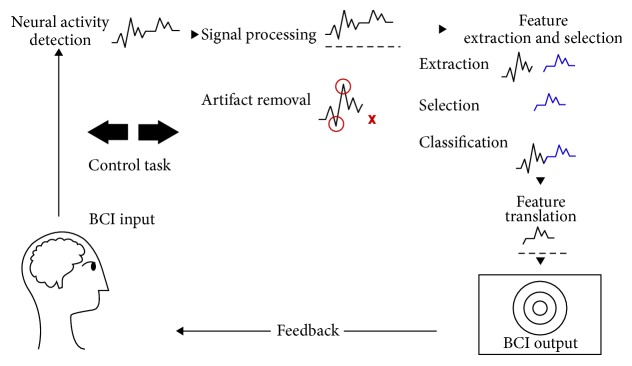
Block diagram of a brain-computer interface system.

BCI is as a nonmuscular communication channel that attempts to reestablish the interaction between an individual and his/her environment. A BCI system involves two stages: calibration (offline analysis) and control (online analysis). The former refers to training processes of a machine to recognize different brain patterns of the user, and the latter concerns the control of a device of interest via the trained machine. The essential function of a BCI is as follows. The user is who controls the device in the system by modifying his/her brain state through external (e.g., visual, auditory, or tactile stimuli) or internal stimulation (e.g., mental tasks). Such brain activity modulation is sensed, amplified, processed, displayed, and saved in two different ways, invasive and noninvasive. The most commonly used invasive recording method is electrocorticography, while some examples of noninvasive methods are electroencephalography (EEG), functional magnetic resonance imaging, and near-infrared spectroscopy. EEG has, however, become the widely used method in BCI community. Once brain signals have been acquired, a feature generator emphasizes relevant neurophysiological features and generates feature vectors in time, frequency, or space domains, or even thereof. The feature translator then attempts to differentiate among control and noncontrol states and translates the classifier output into control commands. The control module and the device controller convert the control commands into semantic control signals for a particular device.  Figure 2 illustrates the structure of BCI systems [28–34].

According to [35], BCI systems can be classified into active, reactive, and passive systems.* Active systems* produce their outputs from commands modulated directly by users in a conscious mental state. The most commonly used control task in active systems is motor imagery (MI), which relies primarily on the detection of slow cortical potentials, sensorimotor rhythms (SMR), and movement-related cortical potentials (MRCP). In particular, SMR can be estimated under two schemes: absolute and relative. In the former case, SMR are not referenced against a baseline state and the processing technique is known as band power. In the latter case, SMR are referenced against a baseline state, typically extracted in a couple of seconds before MI activity, and the processing technique is well-known as event-related (de)synchronization. In both cases, the signal power in *μ* (8–12 Hz) and *β* (16–24 Hz) frequency bands is being quantified.* Reactive BCIs* produce their outputs from reactions to external stimuli such as visual, auditory, and tactile. Most of reactive BCIs rely on the detection of event-related potentials (ERP) that are brain responses, appearing some hundreds of milliseconds after stimulus onset, with different polarities, and at different recording sites. The most widely used ERP is P300, which is a positive potential, appearing from 300 to 500 ms after stimulus onset and frequently over parieto-occipital area. P300 is a component associated with selective attention and memory mechanisms. Other types of reactive BCIs are those based on steady-state evoked potentials, which are much more responsive to sensory input decoding, rather than cognitive processes such as P300. Lastly, in* passive BCIs*, users' mind does not control the system directly as in active and reactive systems. These systems are applied to detect mental workload, working memory load, fatigue, self-induced errors, and deception or anticipation errors (and many other states) when users interact with mobile devices, vehicles, robots, or any other systems.

### 4.2. Relevance of Brain-Computer Interfaces

Although BCI development has been encouraged over the past few years, there is a general lack of research in portable and reliable technology to detect brain activity; accurate and efficient algorithms; direct, relevant, and constructive feedback techniques; and instructive and intuitive interactive methods. According to [32], BCI research should be conducted on the basis of three factors: (1) recent appearance of powerful and inexpensive hardware and software that can perform complex high speed analysis of brain activity, (2) greater understanding of the CNS that has emerged from research, and (3) new recognition of needs and abilities of people suffering from disorders such as cerebral palsy, spinal cord injury, stroke, amyotrophic lateral sclerosis, multiple sclerosis, and muscular dystrophies. BCI progress has always been of particular interest for industrial and medical areas, and applications have been mainly considered in five areas [32]: (1)* replacement*, a BCI may replace CNS function in people with neurodegenerative diseases such as multiple sclerosis; (2)* restorage*, a BCI could restore mobility by reconnecting the peripheral nervous system and the musculoskeletal system in people with amputations; (3)* enhancement*, a BCI might enhance human reactions: for example, it can monitor levels of attention in order to raise alertness when necessary; (4)* supplementation*, a BCI system could supplement natural CNS output: for example, it can be used to control robotic arms as an aid in several tasks ranging from computing to industrial applications; and (5)* improvement*, a BCI can also improve the functionality of devices such as orthoses by monitoring natural CNS outputs and providing feedback that would lead to control properly and effectively the orthosis of interest.

### 4.3. Controversial Issues

Even when promises and expectations on BCIs have increased considerably, these systems are not a completely working prototype. In accordance with [36], BCIs have four potentials pitfalls. Firstly, far too little attention has been paid to end-user requirements when designing BCI solutions, particularly those associated with human aspects, learning strategies, and interactive design. In this respect, it has been well documented that up to 40% of healthy users cannot control an active BCI system at all, while the remaining ones only reach a moderate performance. This phenomenon is called BCI illiteracy and indicates that the omission of end-user needs and their cognitive profiles may be playing a crucial role in BCI shortcomings [37]. Secondly, researchers in the field seem to neglect that user behavior and experience in BCI systems largely depend on coping with the control task, previous sensorimotor abilities, and motivation. As users must produce stable, clear, and detectable neural patterns, training procedures, and feedback methods should facilitate the acquisition of control skills based on modulation of EEG signals. Thirdly, real working environments are much noisier, more dynamic, and unforeseeable in contrast with well-controlled laboratory environments; therefore, signal processing and pattern recognition should be versatile and robust algorithms. Finally, there is a lack of clear metrics to assess the effective performance of a BCI system. It is not clear yet how to weight human and machine factors, such as detection and accuracy, respectively, on metrics that result from BCI outputs. Up to now, researchers in the field have reported metrics directly obtained from the performance of machine learning classifiers, specifically accuracy, and specificity. Nevertheless, the very own nature of classifier metrics cannot indicate whether the user has correctly modulated his/her brain signals or whether he/she is comfortable and concentrated on the control task in use.

### 4.4. How Can VE Improve BCI in Terms of HCI?

Not only is a BCI related to the development of the system per se, but it is also associated with the design of a good quality HCI, considering that BCI users need to be trained exhaustively. The key aspects of user training are repetition, feedback, and motivation [38]. Users must repeat the control tasks over and over since human beings normally learn by trial and error practice. This learning process can be accelerated through feedback and motivation. Feedback provides information about the performance of the ongoing control task, which gradually improves the user performance in the forthcoming repetitions. Motivation creates an encouraging environment, where the growing fatigue caused by the repetitiveness of the control tasks can be reduced. The user training eventually leads to automatizing control tasks, allowing users to confine their attention on the control device, rather than on the function of the BCI system.

The assumption of isolating cognitive processes related to BCI control, along with the disregard of human factors and environment demands (as discussed above), has complicated HCI in BCI applications. In recent years, VEs have become an attractive alternative to enrich HCI in BCI systems. It has been considered that VEs facilitate the user-system adaptation in BCIs because they provide user senses with appropriate feedback. Furthermore, users can learn to control BCI systems under more realistic conditions because virtual simulations offer a more direct interaction with the environment. In general, it has been demonstrated that users are much more comfortable when they manipulate a BCI system in a VE. This is because VEs induce motivation and entertainment, and even more, offer an ample scope on how to achieve a goal [21, 39, 40].

VEs have become a promising alternative to enrich HCI in BCI systems since they lead to a higher user performance [41]; they test BCIs under more realistic situations; they improve attention, motivation, and learning; they facilitate prototyping; and they are feasible for diagnostic and therapeutic purposes [12]. A more detailed account of these points is given hereunder.

#### 4.4.1. Higher BCI Performance

It has been considered that highly immersive VEs induce a high sense of presence, which in turn facilitates BCI performance because VEs provide the user senses with appropriate feedback. A better BCI performance results in a shorter user training and a higher user confidence. VEs could lead to greater performance due to their nature of accurately representing elements of real life in the virtual domain. These representations of environments and objects permit the elaboration of a virtual scenario which can map everyday tasks and routines. This mapping allows establishing a training protocol that can provide feedback associated with the tasks in use. The current interactive systems are not explicit enough to become congruent with the tasks in use. While implementing VEs demands effort and time, often not available, the payoff relies on the possibility of representing and contextualizing tasks for users, who see and become part of something beyond abstract symbols on the screen. In a VE, users can perceive the ongoing changing of their mental tasks. For example, if a mental task is to imagine “kicking a ball,” and then, they see a virtual leg coming from themselves to kick a ball, they will have sense of proprioception and agency. VE offers the possibility of being explicit and accurate. Virtual representations encourage users to generate and maintain mental images by facilitating sensory information and providing feedback within a meaningful context for them [18, 41–45].

#### 4.4.2. BCI Implementation under More Realistic Situations

Human interaction is a huge limitation in laboratories. As virtual simulations offer a more direct interaction with the environment, users can learn to control systems under more realistic situations. Furthermore, the influences of human factors (such as mental fatigue, frustration, or idleness) and distraction sources (such as other people's conversations, ambient noises, or household appliances working) on BCI usability can be studied simultaneously.

The term “realistic situation” does not only refer to high technological implementations, but it also concerns the VE relevance for the users [46]. This factor could even have a higher impact on the system performance. A good example of this is the work presented in [41]. In such work, the control task was to imagine the draw of different basic strokes of Chinese characters. Furthermore, the effectuation of the control task was as real as possible since users observed the explicit representation of the drawing process. Researchers considered that the graphical presentation of imaginary movements could promote MI generation. The research study was conducted as follows. Fourteen subjects (between 22 and 25 years) were divided into two groups: experimental and control. The experimental group used the proposed paradigm based on drawing basic strokes of Chinese characters. The control group used the traditional Graz approach. On average, the experimental group achieved 79.8% system accuracy, whereas the control group yielded around 65.1%. In addition, participants filled in a UX questionnaire, and results suggested that the proposed paradigm was easier to use and more understandable. Overall, this work strengthens the idea that VEs must be contextualized to provide a familiar working environment where users can make full use of their previous knowledge. In this work, it was shown that the modulation of EEG signals through MI activity could be significantly improved if appropriate environmental working conditions are provided.

#### 4.4.3. Improvement of Attention, Motivation, and Learning

Galliard and collaborators (whose work is cited in [47]) defined a human state as the psychophysiological regulation of the brain to reach an optimal condition. This process enables humans to meet environment demands. In this respect, the readiness to catch relevant stimuli (attention) and the desire to learn and to explore (motivation [48]) are essential in BCI applications. VEs have proved to be a potential tool for directing attention, increasing motivation, and accelerating learning of BCI users.

#### 4.4.4. Laboratory for Prototyping BCI Systems

Virtual experiments can facilitate the development of BCI systems, and exhaustive testing of BCI prototypes could be also undertaken. In fact, this might justify the huge expense of implementing physical devices such as robot arms and exoeskeletons.

#### 4.4.5. Diagnostic and Therapeutic Purposes

VEs are suitable for guiding severely paralyzed patients through how to adapt themselves to their new circumstances (e.g., how to control a wheelchair) or on how to regain their basic functions such as walking or talking.

### 4.5. Advances in the Field

A large number of virtual applications in BCI systems have already been undertaken. Active BCIs have been mostly used for navigation purposes [49, 50], and to improve user performance by increasing user motivation [10, 51]. Reactive BCIs have been used to select and manipulate objects inside virtual dwelling places. For example, P300 evoked potentials have been applied to control the functionality of devices such as TV, lamps, or fans [52, 53]. Another example is the utilization of steady-state visual evoked potentials (SSVEPs) to control the behavior of virtual avatars [12, 54]. On simulations of daily applications, VEs and BCIs interactive system have represented scenarios ranging from holding a cup and pouring water [43] to identify and recognize subjects [55]. However, applications have also been focused on more engaging experiences such as playing tennis [39] or even an aesthetic experience provided by a virtual play [56]. Despite the several directions presented on the advances on the intersection between BCI systems and VEs, in further sections trends on this field will be explained and detailed.

In this section, a review about the existing body of research on VE applications in BCIs is presented, excluding those related to gaming purposes. Video games are usually used for entertainment; however, the system contextualization regarding the user requirements is neither specified nor considered. The review presented in this section attempts to highlight the enrichment of BCI systems by means of VEs in terms of human behavior and learning, user adaptability, significance of virtual scenarios, and user concerns. Specifically, all those research studies carried out to facilitate the acquisition of MI skills by providing high quality of immersion and spatial cognition are of special interest. A great deal of research into this framework has focused on augmenting the level of interaction between the user and the system in order to evoke and maintain clearer EEG patterns (e.g., MRCPs and SSVEP), thus increasing the pattern recognition efficiency. Researchers in the field are aware of the importance of using VEs as interactive paradigms for HCI enrichment. Their work has shown that sensory-enriched interfaces, particularly in visual modality, do not only provide satisfactory system outcomes, but they also make users feel comfortable and attentive during the interaction.

It is considered that the user ability to modulate his/her EEG signals by MI can be much more gainful to enhance BCI performance, rather than the computational algorithm complexity. Users have been ignored so far, and possibly if now we pave the way for facilitating human learning and adaptation, they could finally establish a regular communication with the system. In the following sections, three main topics are discussed: (1) VEs as working environments and control panels, (2) VEs for navigation purposes in BCI systems, and (3) relevance of user mental state in sensory-enriched environments. The most purposeful and recent works on this matter are summarized in Table 1.

#### 4.5.1. Working Environments and Control Panels

Virtual reality (VR) and augmented reality (AR) have been widely used in reactive BCIs based on SSVEP since the level of user attention towards visual stimuli increases significantly. In a study conducted in [43], three male subjects aged between 25 and 27 years were asked to perform two types of tasks: VR-based and AR-based. The aim of this study was to assess AR as a means to emulate not controlled environments such as patients' home or hospital. The general task was to navigate across a virtual room and through an avatar. Three participants were recruited for the study and their performances revealed that they had greater difficulty in controlling the avatar in AR mode. Researchers suggested that distracting elements in AR scenarios hindered the avatar manipulation. AR forces users to interact with surroundings at any time, which definitely complicates the interaction between user and system. AR may be harnessed to analyze BCI systems under environments where users' attention, immersion, and performance are compromised by external factors [42].

On the other hand, VR can be applied to get the BCI system under control. By way of illustration, in [61], it was improved the performance of a hybrid BCI by employing VR technology based on Oculus Rift system. The aim of this study was to develop an efficient virtual control panel. The VE consisted of three spheres in different colors on which users must direct their attention. Once users had decided the one to be selected, they must imagine such sphere approaching to them. Attention on the spheres was detected via eye-trackers, but the sphere approximation was quantified by EEG processing. This control mechanism was very efficient because it was natural and intuitive. Users could understand clearly how to control a BCI system, even in a highly demanding situation. It is worth noting that BCI function relies on both user ability (imagination) and technology aspects (eyes' position). This lightened the workload regarding control tasks, and allowed users interact more easily [57].

#### 4.5.2. Navigation Systems

Typically, VEs have been applied to navigate in virtual worlds. Researchers in the field have worked towards two major goals: transportation and effects of vehicular environmental stimuli on human reactions [61–64]. However, the application of navigation systems has recently gone beyond these two purposes. A notable example of this is the work presented in [65], who developed a VE using Oculus Rift system that was controlled through a BCI based on MRCPs. The key aim of this study was the pattern recognition of four different navigational directions (forward, backward, go right, and go left) decoded in MRCPs of the user. Authors demonstrated that VEs are quite efficient to train BCI users and make users generate different EEG patterns for different movements [58]. Another example of the usage of specific potentials include SSVEPs, where the authors have relied on the detection of these potentials in order to select a specific direction for navigating on a virtual environment; rather than using motor imagery, this work relied on eye fixation on four points on the environment representing possible directions of navigation (forward, backward, go right, and go left). They later took advantage of the graphic nature of VEs and the nature of SSVEP for the proposal of a paradigm for navigation using a BCI system which relies on attending key points of a graphic representation of a daily environment [65].

Vehicle control is another representative example of novel application of navigation systems. In [26], a flight simulation system with brain-computer interacting controls was implemented. A 53-year-old woman with quadriplegia was instructed to control a virtual airplane by correlating airplane movements in full flight with her arm movements. Researchers concluded that metaphorical interaction and practice did not lead to one-to-one relationship between arm and airplane movements. Nevertheless, user attention can be confined for longer periods of time, resulting in the mastery of MI based control tasks. The feminine user was able to control the airplane with no restriction after two training sessions. Authors argued that the feedback method in use was sufficiently efficient to instruct user how to modulate her brain signal using her arm movements [26]. In a similar case, in [58], a study based on the detection of pilot induced oscillations susceptibility was conducted. Researchers designed a flight VE with a joystick based control mechanism. Control tasks were based on boundary avoidance task. That is, users required flying the plane on a specific trajectory, and whether they failed to follow the same trajectory, the flight simulation stopped automatically. Results showed that workload buildup in boundary avoidance tasks could be successfully decoded from EEG oscillations in *δ*, *θ*, *α*, *β*, and *γ* frequency bands.

Particularly, *θ* band over frontocentral recording sites and *γ* band over lateralized somatosensory areas were the major contributors in the EEG pattern recognition [64].

Apart from MI activity, other applications of navigation systems have played an important role in BCI research. This can be illustrated in [27], where a VE that rendered driving environments for children with autistic spectrum disorders (ASD) was designed. The virtual system consisted of a car to be driven in a city with full of details in the surroundings, including buildings, trees, pedestrians, and traffic lights. Authors claimed that realistic tasks might stimulate neural processes such as workload management, long-term memory access, visuospatial processing, regulation of emotions and attention, and decision-making, in children suffering from ASD. In this study, authors made use of EEG signals to detect emotions and cognitive states, including concentration, boredom, frustration, and mental load. As system performance was between 78% and 95%, this BCI based on virtual architecture seems to be promising to treat ASD [27]. In the same line of thinking, in [66], an emotion detection based on BCI technology to develop a decision-making system was proposed. Five subjects trained an intelligent agent by reinforcement learning to navigate through a virtual city where decision-making was based on user emotions, rather than user intentions as usual. The VE rendered a car cabin through which users could explore the virtual city. Instead of decoding user intentions, an intelligent agent received BCI outputs concerning human reactions such as surprise, anxiety, happiness, or concentration. All these human reactions were learned by the agent, which controlled the trajectory of the virtual vehicle [59].

Last but not least, navigation through virtual dwelling places has become one of the most examined applications. The work presented in [64] is a good exemplification of HCI enrichment in this type of navigation systems. Those researchers quantified levels of attention in VEs by detecting P3b components. The detection of P3b was based on color coding, and the user propose was to access different rooms in a virtual house. Authors demonstrated that color coding is a more proficient way to capture and hold user attention than the classical Donchin paradigm [63].

#### 4.5.3. User Mental State

User mental state at the moment of the interaction is a key element to reach a stable performance system. According to [66], the modulation of EEG signals using MI activity greatly depends on the user mental balance since control tasks become much more differentiable. This can be seen in [11], where an interactive system based on mindfulness and meditation was designed. By using an Oculus Rift system to render the VE, a Leap Motion system to track hand movements, and a Muse headband to record EEG activity, researchers set up a stimulating environment to practice levitation, pyrokinesis, and telekinesis. Their setup induced great sense of immersion, which, in turn, promoted meditation and mindfulness, which facilitated MI training later [11]. Similarly, in [67], a VE where users controlled an avatar by their levels of concentration was proposed. By employing RelaWorld software and a ERP based BCI, authors significantly improved user-system interaction only prolonging lapse of concentration [60].

#### 4.5.4. Applying VEs to BCI Paradigms

To control a BCI system is a skill that must be acquired. The process of learning in current BCI paradigms generally stimulates only one sensory pathway, either visual or auditory. However, humans gather information from five sensory pathways (vision, hearing, touch, smell, and taste) and react accordingly. It has been shown that if environments are sensorially enriched, learning is much more effective. The effects of environmental enrichment are exemplified in the work reported in [67], where two groups of cortically injured rats were exposed to enriched and nonenriched environments. The enriched environment involved a variety of elements, including group housing, social stimulation, competition for food and water, stress, greater motor activity, manipulation of objects, and sensory stimulation augmentation. The nonenriched environment only involved food and water. The results showed that rats exposed to environmental enrichment made significantly fewer errors in their tasks than those in nonenriched conditions. Furthermore, three neurophysiological modifications were found. First, certain zones of the cerebral cortex, which are used in complex learning and problem solving processes, became heavier, deeper, and greater. Second, the neurons were larger, the synapse to neuron ratio was higher, the synapses were bigger, and there was more profuse dendritic branching in those zones. Third, there were clear effects of enrichment at the level of neurochemistry. An example of this is the considerable augmentation of the RNA/DNA ratio, which indicates an increased metabolic rate. In this work, it was demonstrated that the most important factor for stimulating brain changes was the enforced interaction with enriched environments. On the other hand, it has been found that sensory feedback plays a central role in the human learning process. The human brain makes use of sensory feedback to make predictions, thereby modifying human behaviors [68]. As learning is a process that involves changes in behavior that arise from interaction with the environment, it means that sensory feedback does not only influence behavioral patterns, but it also promotes perceptual learning. Recent neuroimaging evidence suggests that perceptual learning promotes neural plasticity over sensory-motor cortices and increases connectivity between such areas of the brain. Furthermore, the effect of perceptual learning is durable [69, 70]. This means that somatosensory function plays a vital role in human learning. It is hypothesized that if sensory feedback is properly given, perceptual learning will be gained, which in turn will achieve the acquisition of skills to control a BCI system.

In the light of the above information, it is encouraged to take advantage of VE features to provide sensorially enriched environments, which in turn may facilitate the acquisition of skills to control a BCI system. To work towards this goal, the adaptation of VEs via interactive methods for brain-computer communication sounds promising. This requires a process of conceptualization and design, which primarily depends on tasks or actions undertaken by users. The application and integration of VEs along with sensory stimulation in BCI paradigms rely on four stages: context, metaphor, design, and evaluation [71, 72].

*Context.* Considering a VE as an outcome that involves interactive design, earlier studies must be done to discover the correlation between the virtual proposal and a group of items that includes the user context (specifically everyday tasks), working environments, commonly used technology, devices, and navigation. These factors determine a metaphor, which integrates the user context with the set of tasks to be performed in the interactive system. Thereby, a contextualized scenario is constructed. Although HCI community has acknowledged the importance of human factors in the design and conceptualization of interactive systems for several years, the overlook of these factors has not only produced misleading interactive models but also inefficient VEs. The context of BCI systems is important for users since this helps to build awareness about the relevance of BCI training and control. So far, the classical example of contextualized applications is control tasks related to activities of daily living such as turning on and off lamps and switches [25] and wheelchair control [26]. A more recent and notorious example of contextualization is given in [41], where all participants were Chinese and the MI control task was directly associated with activities of their daily living, that is, drawing of basis strokes of Chinese characters. 

*Metaphor.* Once the metaphor is established, the interactive design and layout of the VE can be proposed. Exploiting the metaphor leads to find optimal cues, feedback, and actions to be undertaken inside the VE. It is important to consider interactive design as a heuristic method to find solutions to a specific problem, rather than an ultimate solution. In particular, the metaphor based on concentration and mindfulness provides users with powerful tools to interact with the VE, including higher attention, clearer perception, and better conceptualization [11]. A good example of a movement metaphor was proposed by [39], where the task of hitting a tennis ball in a virtual court was used. In that environment, users could see an explicit outcome of their mental images. In this case, the metaphor was used to stimulate the imagination of a movement towards a specific direction. Another notable example is the metaphor used in [41], where the task of drawing basic strokes of Chinese characters was employed. Similar to [39], users observed the rendering of their imaginary writing. 

*Design.* The overall layout, the model complexity, and the sensorial features depend on context, user profile, and available technological resources. Returning to aesthetic and functional features considered in the context stage, it is essential to design familiar, stimulating, and favorable environments for users. Particularly, details are critical when emulations of real-life situations are attempted. Lack of detail and/or emphasis in design might make users feel indifferent and disinterest. Flight simulators and car navigators are a good picture of interactive design applications, where details enrich beautifully the environment [26, 27, 62, 64]. Another case in point is the one shown in [41]. The black background, along with the animated image of a hand holding a chalk, was a close analogy of writing on a blackboard. This design illustrates the benefits and advantages of VEs in terms of graphic representation. 

*Testing.* The first testing is an opportunity to gather information from potential users about the early version of a virtual implementation, including interaction flow between user and system and feedforward and feedback sources and models. This can come up with relevant interactive and aesthetic redesigns from users' perspective. Major changes based on further testing are advisable. It is essential to go through an iterative process of design, engaging users from the beginning and along the whole process. In each iteration, users' feedback must be taken in account, and, even more, it should be implemented properly. Although this iterative process demands resources and time [36], it could lead to an optimal and complete interaction between brains and machines.

## 5. Conclusion

The first applications of VEs in BCI research concerned the strength of user motivation, the maintenance of attention for longer periods, and the implementation of favorable feedback mechanism. However, virtual technology had been only seen as a tool to render illusory effects of realism by means of 3D graphics and electronically equipped helmets, headphones, goggles, and gloves. At present, tridimensional representations have become an attractive alternative to enrich HCI since they stimulate cognitive processes that take place while the user navigates and explores VEs, which are mainly associated with workload management, long-term memory access, visuospatial processing, regulation of emotions and attention, and decision-making. The evidence presented thus far shows that VEs can set out working environmental conditions, maximize the efficiency of BCI control panels, implement navigation systems based not only on user intentions but also on user emotions, and regulate user mental state to increase the differentiation between control and noncontrol modalities.

## Figures and Tables

**Table 1:** Comparison of recent applications of VEs in BCI systems.

Authors	Type of environment	BCI System	Type of potential searched	Algorithm for detection	Contribution/novelty
Faller et al. 2017 [42]	Avatar navigation with sound stimuli	g.tec biosignal amplifier	SSVEP	Harmonic sum detection (HSD)	Comparison of feedback provided by users using VR and AR
Chun et al. 2016 [57]	Object manipulation	Emotiv EPOC	SSVEP	Common spatial P patterns (CSP) 8–30 Hz and support vector machines (SVMs)	Using concentration as a way to interact with environment
Kryger et al. 2017 [26]	Flight simulation	NeuroPort Neural Signal Processor	SSVEP	—	Mapping of airplane movements (roll, pitch, yaw) to neural commands
Fan et al. 2017 [27]	Flight simulation	Emotiv EPOC	None	*k*-nearest neighbors (kNN)	Measuring emotions with EEG signals along with a VE
Chen et al. 2016 [58]	Wheelchair control simulation	—	MRCP	—	Detection of patterns in MRCP in four different navigational directions.
Shih et al. 2017 [59]	Car driving simulation	—	—	Double deep Q learning	Training of intelligent agent using emotion detection from EEG signals
Amores et al. 2016 [11]	Superpowers' simulation	Muse headband	—	—	Studying levels of concentration in EEG by stimulation with VEs based on mindfulness and hand movement
Yan et al. 2016 [56]	Virtual play and scenario	Emotiv EEG Headset	Amplitudes of *α*, *β*, and *θ* waves	—	Studying levels of concentration present in EEG signals by stimulation with VEs focused on aesthetic experiences
Kosunen et al. 2017 [60]	Meditation simulation with avatar	RelaWorld system	ERPs	—	Studying levels of concentration in EEG by stimulation with VEs based on mindfulness
Yazmir & Reiner 2017 [39]	Tennis game simulation	Biosemi 64 channel EEG recording system	ERPs ERS/ERD	Blind source separation (BSS) 0–50 Hz	Measurement of correlation between success and error peaks presented on ERPs
Cecílio et al. 2016 [54]	Trash separation game	ActiCHamp amplifier	*µ*-rhythms	Independent component analysis (ICA), principal component analysis (PCA) and SVMs	Utilization of a virtual avatar as a representation of desired movement
Herweg et al. 2016 [61]	Wheelchair simulation	g.USBamp	P300	Step-wise linear discriminate analysis (SWLDA) 0.1–30 Hz	Combination of virtual navigation system along with P300 and tactile feedback
Cyrino & Viana 2016 [43]	Daily tasks simulation, filling a bowl with a cup, rotating levels	Emotiv EPOC	—	—	Virtual environments using daily tasks
Liu et al. 2016 [62]	Car driving simulation environment	NeuroScan NuAmps Express system	—	Fuzzy Neural Network (FNN) Delta, theta, beta and alpha channels.	Usage of FNN as a classifier for predicting driving fatigue
de Tommaso et al. 2016 [63]	Virtual home navigation	Micromed System Plus	P300b	ANOVA 0.5–80 Hz	Virtual environment could be personalized with different light/color options in order to look for different stimuli in simulation
Saproo et al. 2016 [64]	Flight simulator	Biosemi B.V. ActiveTwo	—	ICA 1–55 Hz	Generalization of similar control failures in other cases of tight man-machine coupling where gains and latencies in the control system must be inferred and compensated for by the human operators
Chen et al. 2017 [65]	Landscape navigation	BioSemi ActiveTwo	SSVEP	Canonical correlation analysis 1–80 Hz Multiclass LDA	Employment of SSVEP for navigation in virtual environments.
Gordon et al. 2017 [55]	Target recognition	BioSemi ActiveTwo	P300	Convolutional Neural Networks 0.1–50 Hz	Real-time application for performing BCI-based Human-Centric Scene Analysis.
