# Corrigendum to “Enrichment of Human-Computer Interaction in Brain-Computer Interfaces via Virtual Environments”

**DOI:** 10.1155/2018/7129735

**Published:** 2018-09-06

**Authors:** Luz María Alonso-Valerdi, Víctor Rodrigo Mercado-García

**Affiliations:** Escuela de Ingeniería y Ciencias, Tecnológico de Monterrey, Eugenio Garza Sada 2501, 64849 Monterrey, NL, Mexico

In the article titled “Enrichment of Human-Computer Interaction in Brain-Computer Interfaces via Virtual Environments” [1], the first and last names of all the authors were reversed. The revised authors' list is shown above.
